# Testagem Genética na Cardiomiopatia Hipertrófica: Ampliando a Representatividade Global e Refinando a Interpretação

**DOI:** 10.36660/abc.20260157

**Published:** 2026-04-10

**Authors:** Fernando Rabioglio Giugni

**Affiliations:** 1 Hospital das Clínicas da Faculdade de Medicina da Universidade de São Paulo Instituto do Coração Laboratório de Genética e Cardiologia Molecular São Paulo SP Brasil Laboratório de Genética e Cardiologia Molecular - Instituto do Coração do Hospital das Clínicas da Faculdade de Medicina da Universidade de São Paulo, São Paulo, SP – Brasil

**Keywords:** Variantes Genéticas, Cardiomiopatia Hipertrófica, Rendimento Diagnóstico, Sarcômero

A cardiomiopatia hipertrófica (CMH) representa um exemplo paradigmático de como a genética transformou a medicina cardiovascular contemporânea.^1^ Os avanços nas tecnologias de sequenciamento nas últimas duas décadas expandiram substancialmente o diagnóstico molecular, a triagem familiar e as estratégias de estratificação de risco.^2^ No entanto, grande parte das evidências atuais provém de estudos com populações norte-americanas e europeias, com uma representação comparativamente limitada de populações latino-americanas. Nesse contexto, estudos que descrevem as características genéticas e clínicas de pacientes brasileiros são particularmente importantes, tanto para ampliar a compreensão global da CMH quanto para apoiar o desenvolvimento da cardiologia de precisão em diversos sistemas de saúde.

Nesta edição, Beuren et al.^3^ relatam achados genéticos e fenotípicos de pacientes com CMH e seus familiares recrutados em um centro terciário no sul do Brasil. Em consonância com a literatura anterior, variantes em genes sarcoméricos — particularmente MYH7 e MYBPC3 — foram responsáveis por quase metade dos casos. Essas observações reforçam uma mensagem central demonstrada repetidamente em diversas populações: apesar da substancial heterogeneidade genética, a doença sarcomérica permanece o substrato biológico dominante da CMH.^4–6^ É importante ressaltar que estudos conduzidos fora das populações tradicionalmente estudadas são essenciais para garantir a implementação equitativa da genômica cardiovascular e para compreender a potencial variação regional na arquitetura genética.

Além de sua relevância regional, o estudo destaca diversas questões mais amplas que o campo das cardiomiopatias hereditárias enfrenta atualmente.

Em primeiro lugar, a interpretação do rendimento diagnóstico em testes genéticos permanece complexa. As taxas relatadas de variantes patogênicas ou provavelmente patogênicas variam amplamente entre as coortes, influenciadas por padrões de encaminhamento, recrutamento baseado em famílias e critérios de inclusão.^7,8^ O estudo relatou um rendimento de 68%, que excede as taxas tipicamente observadas em coortes populacionais não selecionadas. Estudos que incorporam parentes ou populações com genótipos enriquecidos, como este, podem demonstrar rendimentos aparentes mais altos do que coortes populacionais. Em vez de representar discrepâncias entre estudos, essa variação ressalta como o desenho da coorte influencia fortemente as distribuições genéticas observadas. À medida que os testes genéticos se tornam mais amplamente implementados, estruturas de relato padronizadas — particularmente a distinção entre probandos e membros da família — serão cada vez mais importantes para comparações significativas entre estudos.

Em segundo lugar, as correlações genótipo-fenótipo continuam a representar uma das áreas mais desafiadoras na pesquisa da CMH. Embora investigações anteriores tenham sugerido trajetórias clínicas diferenciais associadas a genes sarcoméricos específicos, os resultados permanecem heterogêneos e frequentemente dependentes do contexto.^4,9^ Tamanhos de amostra pequenos, agrupamento familiar e penetrância incompleta complicam a interpretação, frequentemente limitando as análises a observações exploratórias em vez de conclusões definitivas. O presente estudo relatou uma tendência para maior carga obstrutiva entre pacientes com CMH relacionada ao gene MYH7, enquanto pacientes com variantes do gene MYBPC3 apresentaram uma tendência a arritmias mais frequentes. Isso reflete uma realidade mais ampla: as tendências nas características clínicas entre subgrupos genéticos são intrigantes, mas enfatizam a necessidade de conjuntos de dados colaborativos maiores, capazes de desvendar os efeitos específicos de genes, variantes e ambientes.

Em terceiro lugar, o trabalho ilustra uma tensão persistente entre a expansão dos painéis genéticos e a manutenção da clareza interpretativa. O uso crescente de grandes painéis de sequenciamento inevitavelmente identifica variantes de significado incerto (VUS), o que representa desafios para a translação clínica.^10,11^ O presente estudo identificou 22% de VUS entre as variantes, algumas em genes com associação limitada à CMH. Embora painéis abrangentes maximizem o potencial de descoberta, eles também destacam a importância de estruturas rigorosas de classificação de variantes e da comunicação cuidadosa das incertezas aos pacientes e médicos. O progresso futuro provavelmente dependerá não apenas da escala de sequenciamento, mas também da validação funcional, de bancos de dados de variantes curados e de iniciativas internacionais de compartilhamento de dados.

É importante destacar que o estudo também chama a atenção para as disparidades no acesso a testes genéticos cardiovasculares.^12,13^ Em muitos contextos de baixa e média renda, o custo, as limitações de infraestrutura e a variabilidade na familiaridade dos médicos com a pesquisa genética continuam sendo barreiras à implementação. A população brasileira é caracterizada por um alto grau de miscigenação, o que a torna de grande interesse para estudos genéticos.^14^ Apesar do tamanho modesto da amostra, este estudo contribui para melhorar a representatividade e representa uma importante adição à escassa literatura existente sobre a genética da CMH na população brasileira.^6^ À medida que as recomendações das diretrizes incorporam cada vez mais o genótipo nas estratégias de gestão, o acesso equitativo se torna uma necessidade urgente.^15^

Em resumo, este estudo de coorte brasileiro contribui com dados valiosos de uma população sub-representada e reforça a relevância global dos testes genéticos na CMH. Ao mesmo tempo, destaca os desafios metodológicos e interpretativos que definem a era atual da genômica cardiovascular. Esforços contínuos em direção a estudos colaborativos mais amplos, à padronização dos relatórios e à cuidadosa integração dos achados genéticos na tomada de decisões clínicas serão cruciais para concretizar plenamente a promessa da medicina de precisão na CMH.
