# Supplementary material for: Testagem Genética na Cardiomiopatia Hipertrófica: Ampliando a Representatividade Global e Refinando a Interpretação
Source: Arq Bras Cardiol. 2026 Apr 10;123(2):e20260157. [Article in Portuguese] doi: 10.36660/abc.20260157 (PMC13128186; doi:10.36660/abc.20260157)
Supplement: Translation

# Translation: Genetic Testing in Hypertrophic Cardiomyopathy: Expanding Global Representation While Refining Interpretation

**DOI:** 10.36660/abc.20260157i

**Authors:** Fernando Rabioglio Giugni

**Affiliations:** 1 São Paulo SP Brazil Laboratório de Genética e Cardiologia Molecular - Instituto do Coração do Hospital das Clínicas da Faculdade de Medicina da Universidade de São Paulo, São Paulo, SP – Brazil

**Keywords:** Genetic Variants, Hypertrophic Cardiomyopathy, Diagnostic Yield, Sarcomere

Hypertrophic cardiomyopathy (HCM) represents a paradigmatic example of how genetics has transformed contemporary cardiovascular medicine.^1^ Advances in sequencing technologies over the past two decades have substantially expanded molecular diagnosis, family screening, and risk stratification strategies.^2^ However, much of the current evidence derives from North American and European cohorts, with comparatively limited representation of Latin American populations. In this context, studies describing the genetic and clinical characteristics of Brazilian patients are particularly important, both to broaden global understanding of HCM and to support the development of precision cardiology within diverse healthcare settings.

In this issue, Beuren et al.^3^ report genetic and phenotypic findings from patients with HCM and their relatives recruited at a tertiary center in southern Brazil. Consistent with prior literature, variants in sarcomeric genes—particularly MYH7 and MYBPC3—accounted for nearly half of the cases. These observations reinforce a central message repeatedly demonstrated across populations: despite substantial genetic heterogeneity, sarcomeric disease remains the dominant biological substrate of HCM.^4–6^ Importantly, studies conducted outside traditionally studied populations are essential to ensure equitable implementation of cardiovascular genomics and to understand potential regional variation in genetic architecture.

Beyond its regional relevance, the study highlights several broader issues currently facing the field of inherited cardiomyopathies.

First, the interpretation of diagnostic yield in genetic testing remains complex. Reported rates of pathogenic or likely pathogenic variants vary widely across cohorts, influenced by referral patterns, family-based recruitment, and inclusion criteria.^7,8^ The study reported a yield of 68%, which exceeds rates typically observed in unselected population-based cohorts. Studies incorporating relatives or genotype-enriched populations, such as this, may demonstrate higher apparent yields than population-based cohorts. Rather than representing discrepancies between studies, such variation underscores how cohort design strongly shapes observed genetic distributions. As genetic testing becomes more broadly implemented, standardized reporting frameworks—particularly distinguishing probands from family members—will be increasingly important for meaningful cross-study comparisons.

Second, genotype–phenotype correlations continue to represent one of the most challenging areas in HCM research. Although prior investigations have suggested differential clinical trajectories associated with specific sarcomeric genes, findings remain heterogeneous and often context-dependent.^4,9^ Small sample sizes, familial clustering, and incomplete penetrance complicate interpretation, frequently limiting analyses to exploratory observations rather than definitive conclusions. The current study reported a trend towards higher obstructive burden among MYH7-related HCM, while patients with MYBPC3 variants presented a trend of more frequent arrhythmias. This reflects this broader reality: trends in clinical characteristics across genetic subgroups are intriguing but emphasize the need for larger collaborative datasets capable of disentangling gene-, variant-, and environment-specific effects.

Third, the work illustrates a persistent tension between expanding gene panels and maintaining interpretative clarity. The increasing use of large sequencing panels inevitably identifies variants of uncertain significance (VUS), raising challenges for clinical translation.^10,11^ The current study identified 22% of VUS among variants, some in genes with limited association with HCM. While broad panels maximize discovery potential, they also highlight the importance of rigorous variant classification frameworks and careful communication of uncertainty to patients and clinicians. Future progress will likely depend not only on sequencing scale but also on functional validation, curated variant databases, and international data sharing initiatives.

Importantly, the study also draws attention to disparities in access to cardiovascular genetic testing.^12,13^ In many low- and middle-income settings, cost, infrastructure limitations, and variable clinician familiarity remain barriers to implementation. The Brazilian population is characterized by a high degree of admixture, making it of great interest for genetic studies.^14^ Despite a modest sample size, this study contributes to improving representation and represents an important addition to the sparse existing literature on HCM genetics in the Brazilian population.^6^ As guideline recommendations increasingly incorporate genotype into management strategies, equitable access becomes an urgent need.^15^

In summary, this Brazilian cohort study contributes valuable data from an underrepresented population and reinforces the global relevance of genetic testing in HCM. At the same time, it highlights ongoing methodological and interpretative challenges that define the current era of cardiovascular genomics. Continued efforts toward larger collaborative studies, standardized reporting, and careful integration of genetic findings into clinical decision-making will be crucial to fully realize the promise of precision medicine in HCM.
